# The mutational landscape of *ARMC5* in Primary Bilateral Macronodular Adrenal Hyperplasia: an update

**DOI:** 10.1186/s13023-025-03554-1

**Published:** 2025-02-05

**Authors:** Lucas Bouys, Anna Vaczlavik, Isadora P. Cavalcante, Florian Violon, Anne Jouinot, Annabel Berthon, Patricia Vaduva, Stéphanie Espiard, Karine Perlemoine, Peter Kamenicky, Marie-Christine Vantyghem, Antoine Tabarin, Gérald Raverot, Cristina L. Ronchi, Ulrich Dischinger, Martin Reincke, Maria C. Fragoso, Constantine A. Stratakis, Albain Chansavang, Eric Pasmant, Bruno Ragazzon, Jérôme Bertherat, for the COMETE and ENSAT Networks

**Affiliations:** 1 Université Paris-Cité, Institut Cochin, Inserm U1016, CNRS UMR 8104, Paris, France; 2 Department of Endocrinology and National Reference Center for Rare Adrenal Disorders, Hôpital Cochin, Assistance Publique Hôpitaux de Paris, 27 rue du Faubourg Saint-Jacques, 75014 Paris, France; 3 Department of Endocrinology, Diabetology and Nutrition, CHU Rennes, Rennes, France; 4 Department of Endocrinology, Diabetology, Metabolism and Nutrition, CHU Lille, Inserm U1190, Lille, France; 5 Université Paris-Saclay, Inserm, Physiologie et Physiopathologie Endocriniennes, Department of Endocrinology and Reproduction, Reference Center for Rare Pituitary Diseases, Hôpital Bicêtre, Assistance Publique Hôpitaux de Paris, Le Kremlin-Bicêtre, France; 6 Department of Endocrinology, Diabetology and Nutrition, Hôpital Haut-Lévêque, CHU Bordeaux, Bordeaux, France; 7 Department of Endocrinology, Groupement Hospitalier Est, Hospices Civils de Lyon, Bron, France; 8 Institute of Metabolism and System Research, University of Birmingham, Birmingham, UK; 9 Centre for Endocrinology, Diabetes and Metabolism (CEDAM), Birmingham Health Partners, Birmingham, UK; 10 Division of Endocrinology and Diabetes, Department of Internal Medicine I, University Hospital of Würzburg, Würzburg, Germany; 11 Medizinische Klinik und Poliklinik IV, Klinikum der Universität München, Munich, Germany; 12 Department of Endocrinology, Adrenal Unit, University of Sao Paulo, Sao Paulo, Brazil; 13 Section on Endocrinology and Genetics, Eunice Kennedy Shriver National Institute of Child Health and Human Development, National Institutes of Health (NIH), Bethesda, MD USA; 14 Research Institute, ELPEN, Pikermi, Athens, Greece; 15 Human Genetics and Precision Medicine, IMBB, FORTH, Heraklion, Crete, Greece; 16 Department of Genomic Medicine of Tumors and Cancers, Hôpital Cochin, Assistance Publique Hôpitaux de Paris, Paris, France

**Keywords:** ARMC5, Genetics, Adrenal gland, Cushing’s syndrome, Primary Bilateral Macronodular Adrenal Hyperplasia, Cortisol, ACTH

## Abstract

**Background:**

Primary Bilateral Macronodular Adrenal Hyperplasia (PBMAH) is a rare cause of Cushing’s syndrome due to bilateral adrenocortical macronodules. Germline inactivating variants of the tumor suppressor gene *ARMC5* are responsible for 20–25% of apparently sporadic PBMAH cases and 80% of familial presentations. *ARMC5* screening is now routinely performed for PBMAH patients and families. Based on literature review and own observation, this study aims to give an overview of both published and unpublished *ARMC5* genetic alterations and to compile the available evidence to discriminate pathogenic from benign variants.

**Results:**

146 different germline variants (110 previously published and 36 novel) are identified, including 46% missense substitutions, 45% truncating variants, 3% affecting splice sites, 4% in-frame variants and 2% large deletions. In addition to the germline events, somatic 16p loss-of-heterozygosity and 104 different somatic events are described. The pathogenicity of *ARMC5* variants is established on the basis of their frequency in the general population, in silico predictions, familial segregation and tumor DNA sequencing.

**Conclusions:**

This is the first extensive review of *ARMC5* pathogenic variants. It shows that they are spread on the whole coding sequence. This is a valuable resource for genetic investigations of PBMAH and will help the interpretation of new missense substitutions that are continuously identified.

## Background

Primary Bilateral Macronodular Adrenal Hyperplasia (PBMAH) is an adrenal cause of cortisol excess (Cushing’s syndrome) due to bilateral adrenocortical macronodules. Even though it is the most frequent cause of bilateral adrenal tumors, PBMAH remains an apparently rare, but probably underdiagnosed disease [1]. Rare syndromic presentations of PBMAH can be observed as part of dominantly inherited diseases such as multiple endocrine neoplasia type 1 (MEN1) [2–4], familial adenomatous polyposis (FAP) [5–7], hereditary leiomyomatosis and renal cell cancer (HLRCC) [8, 9], due to germline pathogenic variants of *MEN1* (Menin), *APC* (Adenomatous Polyposis Coli) and *FH* (Fumarate Hydratase) genes, respectively, and others [10]. However, PBMAH presents most frequently (probably more than 95%) in an apparent isolated form, and in this presentation, the bilateral nature of adrenal involvement and the reports of familial cases in the early 1990s [11] led to the suspicion of a genetic predisposition. In 2013, *ARMC5* (Armadillo repeat containing 5) germline heterozygous inactivating variants have been identified in PBMAH patients treated by adrenalectomy [12]. Adrenal nodules harbor a second somatic hit, whether by loss-of-heterozygosity (LOH) or point variation, leading to a bi-allelic inactivation of the gene, which is a common pattern of tumor suppressor genes. In patients with a germline heterozygous mutation of a given tumor suppressor gene (inherited or de novo), the occurrence of a somatic mutation on the other allele of the same gene in a unique cell (an adrenocortical cell in the case of *ARMC5*) leads to the loss of both alleles. This is thought to be the starting point of tumorigenesis in this model. At the time of its identification in PBMAH, *ARMC5*, located at 16p11.2, had never been implicated in human diseases and its function was poorly known. A loss of ARMC5 protein in tumoral tissues with bi-allelic inactivation has been observed [12], driving the development of benign adrenal tumors. Indeed, apoptosis is impaired in cells transiently transfected with *ARMC5* mutated expressing vectors, compared to *ARMC5* wild-type expressing cells [12, 13]. Furthermore, restoration of the apoptosis process after wild-type *ARMC5* re-expression in primary cell cultures from PBMAH patients with *ARMC5* variant has been reported [14]. More recent studies support the involvement of ARMC5 in cell cycle regulation [14] and its turn-over processing by the ubiquitin–proteasome system [15, 16]. Mice knockout for *Armc5* have been engineered. They brought information about ARMC5 implication in early developmental stages, in particular for adrenal glands [17], and its implication in lymphocytes growth and differentiation [18].

Since the initial 2013 report, *ARMC5* germline sequencing is routinely offered in our center to PBMAH patients and to all first-degree relatives of *ARMC5* pathogenic variant carriers. Sporadic (i.e. apparently not familial) PBMAH usually presents with mild hypercortisolism [19], but germline *ARMC5* pathogenic variants, responsible for around 20 to 25% of PBMAH index-cases, are associated with a more pronounced phenotype than observed in wild-type patients, in terms of cortisol secretion, adrenal morphology and complications of Cushing’s syndrome [13, 20]. *ARMC5* variants have also been related to the occurrence of meningioma in some reports [21–27]. A recent study by our group stated that *ARMC5* genotyping should be conditional to the clear presence of bilateral adrenal macronodules (regardless of the number of nodules or size of the adrenals), associated with at least a mild autonomous cortisol secretion, defined by a plasma cortisol after overnight 1 mg dexamethasone suppression test (DST) above 50 nmol/L [20].

Results of *ARMC5* sequencing have also been reported in patients with different phenotypes of the large spectrum of the disease, sometimes incompletely satisfying PBMAH definitions and also, for academic purpose, in patients presenting with adrenal benign nodules in other contexts (MEN1, primary hyperaldosteronism, adrenal incidentalomas). The germline *ARMC5* screening in patients with neuroendocrine tumors (sporadic or associated with MEN1) retrieved germline variants that were at this time classified as likely pathogenic [28]. However, in the light of the most recent data about these variants, all seem to be benign polymorphisms, synonymous variants, or missense variants of uncertain significance, for which the pathogenic nature is unlikely. In the same series, somatic *ARMC5* variants and 16p LOH have been identified in adrenal and extra-adrenal tumors from MEN1 patients without germline *ARMC5* variant, and sometimes LOH and point variants were observed in the same tissue [28]. Besides, the systematic *ARMC5* germline screening in 56 patients with primary aldosteronism identified possibly deleterious variants in 6 unrelated African-American patients [29]. There was no difference regarding the levels of cortisol or aldosterone secretion between variant carriers and wild-type patients, and no second hit has been found in the adrenal tissue of 2 operated variant carriers. Additionally, no *ARMC5* variant has been found in an independent series of patients with primary aldosteronism and bilateral adrenal hyperplasia [30], nor in a series of 4 families with familial hyperaldosteronism type II [31]. At present, there is no strong evidence for a direct relation between *ARMC5* inactivation and mineralocorticoid excess. Recently, a group reported the results of a systematic germline genetic screening of patients with adrenocortical carcinoma (ACC), including *ARMC5* sequencing [32]. Germline variants of *ARMC5* were found in 5 of 150 patients with ACC, these variants are likely benign (p.Thr9Met and p.Pro731Arg) or of uncertain significance (p.Ala23del). No malignant evolution of PBMAH has ever been documented in *ARMC5* variant carriers, so the causative role of these variants in ACC is very unlikely. To date, we cannot affirm the involvement of *ARMC5* in any adrenal condition other than PBMAH.

Numerous *ARMC5* variants have been reported at present, including nearly 50% of missense variants, for which the pathogenic nature is often uncertain. This study aims to give an overview of *ARMC5* alterations already published and recently identified in our center and to propose clues to discriminate pathogenic variants (mutations) from non-pathogenic variants (benign polymorphisms).

## Variants

We performed a systematic literature review for compilation of reported *ARMC5* variants by a search of papers on PubMed and MEDLINE databases, reported from inception of the databases and October 1st, 2024, using the term “ARMC5”. Unpublished variants recently identified in PBMAH patients genotyped in our center were also included in the analysis. These variants were identified by Sanger sequencing as previously described [12] or by next generation sequencing using IonTorrent and Illumina technologies (genomic platform of the Cochin Institute and unit of Oncogenetics of the Cochin hospital) after patient written informed consent for genetic analysis and its research use according to the national regulation. Sequence alignment, variant calling, and variant annotation were performed using MOABI Leaves pipeline (APHP). An assessment of variants’ pathogenicity was performed according to the American College of Medical Genetics and Genomics and the Association for Molecular Pathology (ACMG-AMP) guidelines [33]. Variants were classified in one of the five classes given below: class 1: benign variant, class 2: likely benign variant, class 3: variant of uncertain significance (VUS), class 4: likely pathogenic variant, and class 5: pathogenic variant. Assessment of variants implication was mainly performed based on population databases (gnomAD, v2.1.1, https://gnomad.broadinstitute.org/), variant databases (ClinVar), in silico prediction softwares, familial segregation, and tumoral DNA analysis.

Variants identified in germline DNA were differentiated from those identified in DNA tumor samples. The clinical context was briefly described to distinguish variants identified in PBMAH (and with associated meningioma where appropriate) patients from variants identified in other types of adrenal tumors. To highlight recurrent variants affecting one position, we did not take into account the variants cited repeatedly by a same team, with the exception when unrelated patients were reported. Familial presentations of the disease, usually known before genetic screening, were documented when provided. Somatic variants were listed, and associated germline events were specified if known. All the variants affecting coding exonic sequences and splice sites for canonical transcript NM_001105247.1 were collected. HGVS nomenclature was verified using Mutalyzer online tool (https://mutalyzer.nl/). We used Homo sapiens (human) genome assembly GRCh37 (hg19). In silico prediction synthesis was performed for each variant using Varsome (https://varsome.com/).

### Germline *ARMC5* alterations

**Fig. 1:**
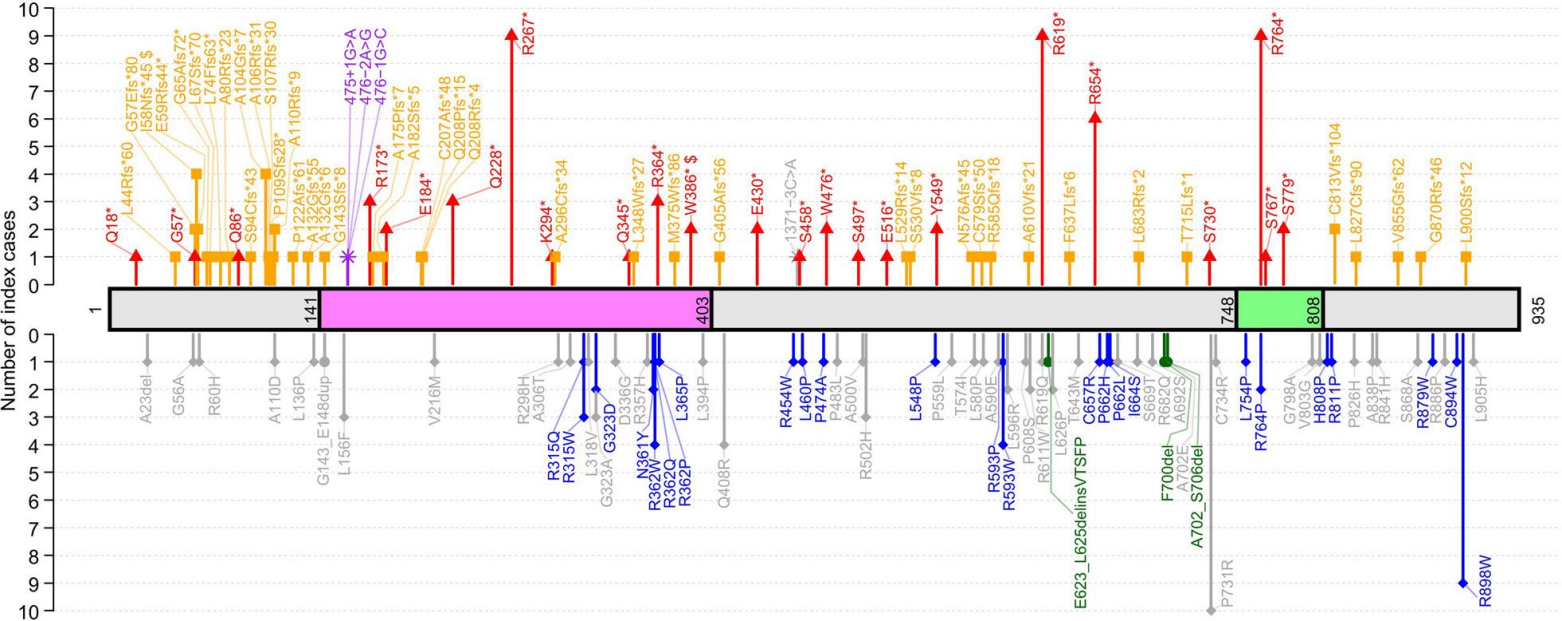
Germline *ARMC5* variants. *ARMC5* variants are shown on a graphical representation of ARMC5 protein from 1 to 935 amino acids (NM_001105247.1 canonical transcript). Both Armadillo and BTB/POZ domains appear respectively as pink and green regions. The class 4 (likely pathogenic) and class 5 (pathogenic) variants are displayed in colors: non-sense (red), frameshift (orange), splice sites (purple), missense (blue) and in frame deletions or insertions (green). The class 2 (likely benign) and class 3 (uncertain significance) variants are represented in grey. Tail size of the lollipops represents the recurrence of each protein alteration from 1 to 10

**Fig. 2:**
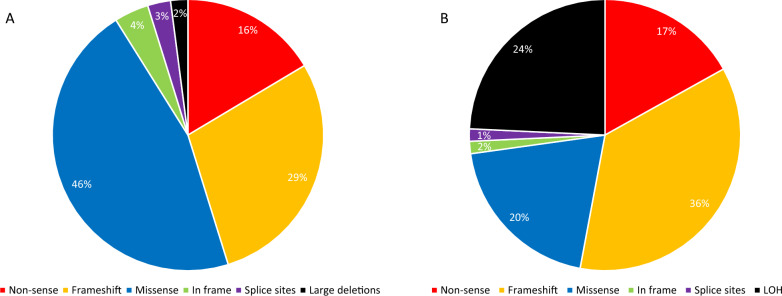
Proportions of protein change categories in germline (panel **A**) and somatic (panel **B**) *ARMC5* likely pathogenic and pathogenic variants

A total of 146 different germline variations of *ARMC5* have been observed in 232 unrelated index patients (including 214 patients meeting diagnostic criteria for PBMAH [20], and 18 investigated for other adrenal conditions) (Table 1), including 110 already reported variants and 36 novel variants identified in our center and not published so far. This list excludes the 5’ and 3’ UTR alterations, intronic variants (outside of the canonical sites) and synonymous variants (with an in silico prediction of not affecting splicing). We also describe three very frequent benign polymorphisms: p.(Phe14Tyr), p.(Ile170Val) and p.(Pro507Leu) (minor allele frequency>0.02 in general population according to gnomAD). Among the 146 germline variants, three were large deletions: a complete *ARMC5* deletion [12], a deletion of 5’UTR and exons 1 to 3 [34], and a deletion of exons 1 to 5 [35]. Interestingly, large deletions were rare and represented only ~ 2% of reported *ARMC5* alterations. The other germline pathogenic variants were 95 single nucleotide variations (SNV) and 48 indel (small insertions or deletions). In total, 67 of the variants were missenses, 42 caused a frameshift, 24 were nonsenses, 4 variants altered the splice sites, 6 were in frame insertions or deletions and 3 were large deletions (Fig. 1 and 2A). These reported genetic alterations affected the entire *ARMC5* coding sequence, and most of them were private, without clear mutational hotspot (Fig. 1 and Table 1). Nevertheless, thirty-four protein variants have been identified in at least two unrelated patients. Indeed, p.(Pro731Arg) has been described in 10 unrelated patients; p.(Arg267*), p.(Arg619*), p.(Arg764*) and p.(Arg898Trp) in 9 patients; p.(Arg654*) in 6 patients; p.(Ile58Asnfs*45), p.(Ala104Glyfs*7), p.(Arg362Trp), p.(Gln408Arg) and p.(Arg593Trp) in 4 patients; p.(Arg173*), p.(Gln228*), p.(Arg315Trp), p.(Gly323Ala), p.(Arg364*), p.(Arg502His) and p.(Tyr549*) in 3 patients; p.(Gly57Glufs*80), p.(Glu59Argfs44*), p.(Ala80Argfs*23), p.(Ala110ArgProfs*9), p.(Glu184*), p.(Gly323Asp), p.(Asn361Tyr), p.(Trp386*), p.(Glu430*), p.(Trp476*), p.(Leu596Arg), p.(Arg611Trp), p.(Leu626Pro), p.(Arg764Pro), p.(Ser779*) and p.(Cys813Valfs*104) in 2 patients. It should be noticed that two of these protein variants were encoded by two different genomic variations: p.(Ile58Asnfs*45) due to either c.170dup or c.172dup and p.(Trp386*) due to either c.1157G>A or c.1158G>A (Table 1). Additionally, some amino acids seem to be more frequently affected by different genetic variations: several different amino acid substitutions affect positions 315, 323, 362, 593 and 662; both missense and nonsense variants can occur at position 619 and 764; amino acid positions 132, 143, 208 are subject to different types of frameshift variations; both frameshift and nonsense variants affect position 57.

**Table 1:** List of the 146 different germline *ARMC5* variants (and 3 benign polymorphisms frequent in the general population), providing the evaluation of their pathogenic nature, based on the criteria according to Richards et al., Genet Med 2015 [33]

HGVS cDNA NM_001105247.1	HGVS Protein	Impact	Classification	ACMG criteria [33]											Somatic 2nd hit	Functional data	Observations	References
c.26C>T	p.(Thr9Met)	Missense	Likely benign	BP3	BP4		PM2										ACC	[32]
c.41 T>A	p.(Phe14Tyr)	Missense	Benign	BA1	BP4		BP3		BP6								frequent benign poymorphism	
c.52C>T	p.(Gln18*)	Nonsense	Pathogenic	PVS1		PM2		PP4										[42]
c.68_70del	p.(Ala23del)	In frame deletion	VUS	PM2													ACC	[32]
c.127_130dup	p.(Leu44Argfs*60)	Frameshift	Pathogenic	PVS1		PM2		PP4										NEW
c.170del (= c.165del)	p.(Gly57Glufs*80)	Frameshift	Pathogenic	PVS1		PM2		PP4										[12, 13, 20]
c.167G>C	p.(Gly56Ala)	Missense	Likely benign	BP4		BP6		PM2		PP4								[37]
c.169G>T	p.(Gly57*)	Nonsense	Pathogenic	PVS1		PM2		PP4										NEW
c.170dup	p.(Ile58Asnfs*45)	Frameshift	Pathogenic	PVS1		PM2		PP4										[14, 24, 81]
c.172dup	p.(Ile58Asnfs*45)	Frameshift	Pathogenic	PVS1		PM2		PP4										[24, 36, 44]
c.174dup	p.(Glu59Argfs44*)	Frameshift	Pathogenic	PVS1		PM2		PP4										[37, 82]
c.179G>A	p.(Arg60His)	Missense	VUS	PM2		PP4												NEW
c.194del	p.(Gly65Alafs72*)	Frameshift	Pathogenic	PVS1		PM2		PP5		PS3		PP4			yes			[37]
c.198del	p.(Leu67Serfs*70)	Frameshift	Pathogenic	PVS1		PM2		PP4										NEW
c.220_222delinsTT	p.(Leu74Phefs63*)	Frameshift	Pathogenic	PVS1		PM2		PP4										[37]
c.237_238insC	p.(Ala80Argfs*23)	Frameshift	Pathogenic	PVS1		PM2		PP4										[20]
c.256C>T	p.(Gln86*)	Nonsense	Pathogenic	PVS1		PS3		PM2		PP4					yes			[12, 13, 20]
c.281del	p.(Ser94Cysfs*43)	Frameshift	Pathogenic	PVS1		PM2		PP4							no			[44]
c.286_310dup	p.(Ala104Glyfs*7)	Frameshift	Pathogenic	PVS1		PM2		PP4										[12, 13, 20]
c.315del	p.(Ala106Argfs*31)	Frameshift	Pathogenic	PVS1		PM2		PP4										[13, 20]
c.318del	p.(Ser107Argfs*30)	Frameshift	Pathogenic	PVS1		PM2		PP4										[38]
c.325_326delinsT	p.(Pro109Serfs28*)	Frameshift	Pathogenic	PVS1		PM2		PP4										[37]
c.327dup	p.(Ala110Argfs*9)	Frameshift	Pathogenic	PVS1		PM2		PS3		PP4					yes		meningioma	[13, 22, 23]
c.329C>A	(p.Ala110Asp)	Missense	Likely benign	PM2		BP1		BP4									myelolipoma	[83]
c.363_373del	p.(Pro122Alafs*61)	Frameshift	Likely pathogenic	PVS1		PM2												[84]
c.394dup	p.(Ala132Glyfs*55)	Frameshift	Pathogenic	PVS1		PM2		PP4										NEW
c.393_394dup	p.(Ala132Glyfs*6)	Frameshift	Pathogenic	PVS1		PM2		PP4										NEW
c.407 T>C	p.(Leu136Pro)	Missense	VUS	PM2		PP3		PP4									meningioma	[27, 44]
c.427_454del	p.(Gly143Serfs*8)	Frameshift	pathogenic	PVS1		PM2		PP4										[40]
c.423_440dup	p.(Gly143_Glu148dup)	In frame insertion	VUS	PM2		PP3		PP4		PM4								NEW
c.466C>T	p.(Leu156Phe)	Missense	Likely benign	BS1		BP4		BP6							no			[20, 28, 29, 39]
c.475 + 1G>A		Splice	Pathogenic	PVS1		PM2		PP4										NEW
c.476-2A>G		Splice	Pathogenic	PVS1		PM2		PP4										[20]
c.476-1G>C		Splice	Pathogenic	PVS1		PM2		PS3		PP4					yes		meningioma	[14, 24, 27]
c.508A>G	p.(Ile170Val)	Missense	Benign	BS1		BP4		BP6									frequent benign polymorphism	
c.517C>T	p.(Arg173*)	Nonsense	Pathogenic	PVS1		PM2		PS3		PP4					yes			[20, 39, 41, 85, 86]
c.523del	p.(Ala175Profs*7)	Frameshift	Pathogenic	PVS1		PM2		PP4										[38]
c.543dup	p.(Ala182Serfs*5)	Frameshift	Pathogenic	PVS1		PM2		PP4										NEW
c.550G>T	p.(Glu184*)	Nonsense	Pathogenic	PVS1		PM2		PP4										[20]
c.618del	p.(Cys207Alafs*48)	Frameshift	Pathogenic	PVS1		PM2		PP4										NEW
c.622dup	p.(Gln208Profs*15)	Frameshift	Pathogenic	PVS1		PM2		PP4										[38]
c.603_622dup	p.(Gln208Argfs*4)	Frameshift	Pathogenic	PVS1		PM2		PP4										NEW
c.646G>A	p.(Val216Met)	Missense	VUS	PP4														NEW
c.682C>T	p.(Gln228*)	Nonsense	Pathogenic	PVS1		PM2		PP5		PS3		PP4			yes			[34, 87, 88]
c.799C>T	p.(Arg267*)	Nonsense	Pathogenic	PVS1		PM2		PS3		PP4					yes		meningioma	[12–14, 20, 21, 26]
c.880A>T	p.(Lys294*)	Nonsense	Pathogenic	PVS1		PM2		PP4										NEW
c.885_886del	p.(Ala296Cysfs*34)	Frameshift	Pathogenic	PVS1		PM2		PP4										[13]
c.893G>A	p.(Arg298His)	Missense	VUS	PM2		PP4												NEW
c.916G>A	p.(Ala306Thr)	Missense	Likely benign	BP4		PP4												[20]
c.943C>T	p.(Arg315Trp)	Missense	Pathogenic	PM2		PM5		PP3		PP5		PS3		PP4	yes	yes		[13, 20, 21, 89]
c.944G>A	p.(Arg315Gln)	Missense	Likely pathogenic	PM2		PM5		PP3		PP5		PP4						[81]
c.952C>G	p.(Leu318Val)	Missense	Likely benign	PM2		PP4		BS3							no			[24]
c.968G>C	p.(Gly323Ala)	Missense	Likely benign	BP4		BP6												[28, 29, 44]
c.968G>A	p.(Gly323Asp)	Missense	Likely pathogenic	PM2		PP4		PS3										[44, 82]
c.1007A>G	p.(Asp336Gly)	Missense	VUS	PM2		BP4												[28]
c.1033C>T	p.(Gln345*)	Nonsense	Pathogenic	PVS1		PM2		PP4										NEW
c.1042del	p.(Leu348Trpfs*27)	Frameshift	Pathogenic	PVS1		PM2		PP4										[37]
c.1070G>A	p.(Arg357His)	Missense	VUS	PM2		PP3		PP4										NEW
c.1081A>T	p.(Asn361Tyr)	Missense	Likely pathogenic	PM2		PP3		PS3		PP4					yes			[46]
c.1084C>T	p.(Arg362Trp)	Missense	Pathogenic	PM2		PM5		PP3		PP5		PS3		PP4	yes		meningioma	[27, 37, 39, 41, 44, 81]
c.1085G>A	p.(Arg362Gln)	Missense	Likely pathogenic	PM2		PM5		PP3		PP4								[40]
c.1085G>C	p.(Arg362Pro)	Missense	Likely pathogenic	PM2		PM5		PP3		PP4		PP1						[20]
c.1090C>T	p.(Arg364*)	Nonsense	Pathogenic	PVS1		PM2		PP5		PS3		PP4			yes			[37, 39, 81]
c.1094 T>C	p.(Leu365Pro)	Missense	Pathogenic	PM2		PM5		PP3		PS3		PP4		PP1	yes		meningioma	[24, 27, 44]
c.1123del	p.(Met375Trpfs*86)	Frameshift	Likely pathogenic	PVS1		PM2												[34]
c.1157G>A	p.(Trp386*)	Nonsense	Pathogenic	PVS1		PM2		PS1		PP4								NEW
c.1158G>A	p.(Trp386*)	Nonsense	Pathogenic	PVS1		PM2		PS1		PP4					yes		meningioma	[14, 24, 27]
c.1181 T>C	p.(Leu394Pro)	Missense	VUS	PM2		PP3		PP4										[24]
c.1214del	p.(Gly405Alafs*56)	Frameshift	Pathogenic	PVS1		PM2		PP4										[38]
c.1223A>G	p.(Gln408Arg)	Missense	Likely benign	BS2		BP6		PS3							yes			[28, 39, 81]
c.1288G>T	p.(Glu430*)	Nonsense	Pathogenic	PVS1		PM2		PP4										[13, 20]
c.1360C>T	p.(Arg454Trp)	Missense	Likely pathogenic	PS3		PP5		PP4										[20]
c.1371-3C>A		Splice	Likely benign	BS1		BP4												[29]
c.1373C>A	p.(Ser458*)	Nonsense	Pathogenic	PVS1		PM2		PP4										NEW
c.1379 T>C	p.(Leu460Pro)	Missense	Likely pathogenic	PM2		PP3		PS3		PP4		PP1			yes		meningioma	[25]
c.1420C>G	p.(Pro474Ala)	Missense	Likely pathogenic	PM1		PM2		PP3		PP4								[20]
c.1428G>A	p.(Trp476*)	Nonsense	Pathogenic	PVS1		PM2		PP4										[20, 45]
c.1448C>T	p.(Pro483Leu)	Missense	Likely benign	BP4		BP6		PP4										[37]
c.1490C>A	p.(Ser497*)	Nonsense	Pathogenic	PVS1		PM2		PP4										[20]
c.1499C>T	p.(Ala500Val)	Missense	VUS	BP4														[28]
c.1505G>A	p.(Arg502His)	Missense	Likely benign	BP4		BP6		BS3							no			[23, 90]
c.1520C>T	p.(Pro507Leu)	Missense	Benign	BS1		BP4		BP6									frequent benign polymorphism	
c.1546G>T	p.(Glu516*)	Nonsense	Pathogenic	PVS1		PM2		PP4										NEW
c.1586dup	p.(Ser530Valfs*8)	Frameshift	Likely pathogenic	PVS1		PM2												[34]
c.1586_1589del	p.(Leu529Argfs*14)	Frameshift	Pathogenic	PVS1		PM2		PP4										[20]
c.1643 T>C	p.(Leu548Pro)	Missense	Likely pathogenic	PS3		PM2		PP4		PP3					yes	yes		[12, 13, 20]
c.1647C>G	p.(Tyr549*)	Nonsense	Pathogenic	PVS1		PM2		PP4										NEW
c.1676C>T	p.(Pro559Leu)	Missense	Likely benign	BP6														[28]
c.1721C>T	p.(Thr574Ile)	Missense	VUS	PM2		PP3		PP4										NEW
c.1726_1753del	p.(Asn576Alafs*45)	Frameshift	Likely pathogenic	PVS1		PM2												[91]
c.1736_1739del	p.(Cys579Serfs*50)	Frameshift	Pathogenic	PVS1		PM2		PP4										[39, 81]
c.1739 T>C	p.(Leu580Pro)	Missense	VUS	PM2		PP3		PP5		PP4								[37]
c.1754_1755del	p.(Arg585Glnfs*18)	Frameshift	Pathogenic	PVS1		PM2		PP4										[20]
c.1769C>A	p.(Ala590Glu)	Missense	VUS	PM2		PP4												NEW
c.1777C>T	p.(Arg593Trp)	Missense	Likely pathogenic	PM2		PP3		PP5		PS3		PP4			yes	yes		[20, 21, 65]
c.1778G>C	p.(Arg593Pro)	Missense	Likely pathogenic	PM2		PM5		PP4		PP5								[90]
c.1787 T>G	p.(Leu596Arg)	Missense	VUS	PM2		PP3		PP4										[82]
c.1822C>T	p.(Pro608Ser)	Missense	VUS	PM2		BP4		PP4										NEW
c.1827_1828dup	p.(Ala610Valfs*21)	Frameshift	Pathogenic	PVS1		PM2		PP4										[20, 39]
c.1831C>T	p.(Arg611Trp)	Missense	VUS	PM2														[28]
c.1855C>T	p.(Arg619*)	Nonsense	Pathogenic	PVS1		PM2		PS3		PP4					yes			[12, 13, 20, 38, 40, 92]
c.1856G>A	p.(Arg619Gln)	Missense	VUS	PP4														[90]
c.1868_1874delins TCACAAGCTTTCC	p.(Glu623_Leu625delins ValThrSerPhePro)	In frame indel	Likely pathogenic	PM2		PM4		PP3		PP4								NEW
c.1877 T>C	p.(Leu626Pro)	Missense	VUS	PM2		PP3		PP4										[43]
c.1908del	p.(Phe637Leufs*6)	Frameshift	Pathogenic	PVS1		PM2		PP4										NEW
c.1928C>T	p.(Thr643Met)	Missense	Likely benign	BS3											no			[29, 39, 81]
c.1960C>T	p.(Arg654*)	Nonsense	Pathogenic	PVS1		PM2		PS3		PP4					yes		meningioma	[20, 27, 40, 44, 89]
c.1969 T>C	p.(Cys657Arg)	Missense	Pathogenic	PM2		PM5		PP3		PP5		PS3		PP4	yes	yes		[13]
c.1985C>A	p.(Pro662His)	Missense	Likely pathogenic	PM2		PP3		PS3		PP4					yes			NEW
c.1985C>T	p.(Pro662Leu)	Missense	Likely pathogenic	PM2		PP3		PM5		PP4							meningioma	[20, 27]
c.1991 T>G	p.(Ile664Ser)	Missense	Likely pathogenic	PM2		PP3		PP5		PS3		PP4			yes	yes		[13, 20]
c.2005 T>A	p.(Ser669Thr)	Missense	VUS	PM2		BP4		PP4										NEW
c.2045G>A	p.(Arg682Gln)	Missense	Likely benign	BP1		BP3		BP4										[28]
c.2048_2060del	p.(Leu683Argfs*2)	Frameshift	Likely pathogenic	PVS1_S		PM2		PP4										NEW
c.2074G>T	p.(Ala692Ser)	Missense	VUS	BP4		PP4												[20]
c.2097_2099del	p.(Phe700del)	In frame deletion	Likely pathogenic	PM2		PM4		PS3		PP4					yes			[13]
c.2105C>A	p.(Ala702Glu)	Missense	VUS	PM2		PP1		PP5		BP4								[93]
c.2104_2118del	p.(Ala702_Ser706del)	In frame deletion	Likely pathogenic	PM2		PM4		PS3		PP4					yes			[12, 13, 20]
c.2139del	p.(Thr715Leufs*1)	Frameshift	Likely pathogenic	PVS1_S		PM2		PP4										[21]
c.2149C>A	p.(Ser730*)	Nonsense	Likely pathogenic	PVS1_S		PM2		PP4										[38]
c.2192C>G	p.(Pro731Arg)	Missense	Likely benign	BS1		BP4		PP5									ACC	[20, 28, 32, 34, 37, 44]
c.2200 T>C	p.(Cys734Arg)	Missense	VUS	PM2		BP4											pituitary adenoma	[94]
c.2261 T>C	p.(Leu754Pro)	Missense	Pathogenic	PM1		PM2		PP3		PP5		PS3		PP4	yes	yes		[13, 20]
c.2290C>T	p.(Arg764*)	Nonsense	Likely pathogenic	PVS1_S		PM2		PP5		PP4								[13, 20, 37, 38]
c.2291G>C	p.(Arg764Pro)	Missense	Likely pathogenic	PM1		PM2		PP3		PP4								[20]
c.2300C>A	p.(Ser767*)	Nonsense	Likely pathogenic	PVS1_S		PM2		PP4										[20]
c.2336C>G	p.(Ser779*)	Nonsense	Likely pathogenic	PVS1_S		PM2		PP4										[24]
c.2393G>C	p.(Gly798Ala)	Missense	Likely benign	PM1		BS1		BP4		BP6								[29]
c.2408 T>G	p.(Val803Gly)	Missense	VUS	PM1		PM2		PP4										NEW
c.2423A>C	p.(His808Pro)	Missense	Pathogenic	PM1		PM2		PP5		PS3		PP4		PP1	yes			[14, 24]
c.2432G>C	p.(Arg811Pro)	Missense	Likely pathogenic	PM1		PM2		PP5		PS3								[82]
c.2436del	p.(Cys813Valfs*104)	Frameshift	Pathogenic	PVS1_S		PM2		PS3		PP4					yes			[34, 95]
c.2477C>A	p.(Pro826His)	Missense	VUS	PM2		BP4												[29, 81]
c.2479del	p.(Leu827Cysfs*90)	Frameshift	Likely pathogenic	PVS1_S		PM2		PP4										[20]
c.2512G>C	p.(Ala838Pro)	Missense	VUS	PM2		PP4												NEW
c.2522G>A	p.(Arg841His)	Missense	Likely benign	PM2		PP3		PP4		BS3					no			NEW
c.2564del	p.(Val855Glyfs*62)	Frameshift	Likely pathogenic	PVS1_S		PM2		PP4										[38]
c.2602 T>G	p.(Ser868Ala)	Missense	VUS	PM2		BP4		PP4										NEW
c.2604_2607del	p.(Gly870Argfs*46)	Frameshift	Likely pathogenic	PVS1_S		PM2		PP4										[20]
c.2635C>T	p.(Arg879Trp)	Missense	Likely pathogenic	PS3		PM2		PP4							yes			[34]
c.2657G>C	p.(Arg886Pro)	Missense	VUS	PM2		PP4												NEW
c.2682C>G	p.(Cys894Trp)	Missense	likely pathogenic	PM1		PM2		PP3		PP4								[20]
c.2692C>T	p.(Arg898Trp)	Missense	Likely pathogenic	PS3		PM2		PP5		PP4					yes	yes		[12, 13, 20, 29, 39, 40, 44]
c.2697dupG	p.(Leu900Serfs*12)	Frameshift	Likely pathogenic	PVS1_S		PM2		PP4										NEW
c.2714T>A	p.(Leu905His)	Missense	VUS	PM2		PP4												NEW
*ARMC5* complete deletion		Large deletion	Pathogenic	PVS1		PM2		PP4										[12]
*ARMC5* 5’UTR + exons 1–3 deletion		Large deletion	Pathogenic	PVS1		PM2		PP4										[34]
*ARMC5* exons 1–5 deletion		Large deletion	Pathogenic	PVS1		PM2		PP4										[35]

When available, the presence of a somatic tumoral second hit or functional data is mentioned. VUS, variant of uncertain significance. List of the referred ACMG criteria: BA1: Allele frequency is>5% in Exome Sequencing Project, 1000 Genomes Project, or Exome Aggregation Consortium; BS1: Allele frequency is greater than expected for disorder; BS2: Observed in a healthy adult individual for a recessive (homozygous), dominant (heterozygous), or X-linked (hemizygous) disorder, with full penetrance expected at an early age; BS3: Well-established in vitro or in vivo functional studies show no damaging effect on protein function or splicing; BP1: Missense variant in a gene for which primarily truncating variants are known to cause disease; BP3: In-frame deletions/insertions in a repetitive region without a known function; BP4: Multiple lines of computational evidence suggest no impact on gene or gene product; BP6: Reputable source recently reports variant as benign, but the evidence is not available to the laboratory to perform an independent evaluation; PVS1: Null variant (nonsense, frameshift, canonical ± 1 or 2 splice sites, initiation codon, single or multiexon deletion) in a gene where loss of function is a known mechanism of disease; PS1: Same amino acid change as a previously established pathogenic variant regardless of nucleotide change; PS3: Well-established in vitro or in vivo functional studies supportive of a damaging effect on the gene or gene product; PM1: Located in a mutational hot spot and/or critical and well-established functional domain (e.g., active site of an enzyme) without benign variation; PM2: Absent from controls (or at extremely low frequency if recessive) in Exome Sequencing Project, 1000 Genomes Project, or Exome Aggregation Consortium; PM4: Protein length changes as a result of in-frame deletions/insertions in a non-repeat region or stop-loss variants; PM5: Novel missense change at an amino acid residue where a different missense change determined to be pathogenic has been seen before; PP1: Cosegregation with disease in multiple affected family members in a gene definitively known to cause the disease; PP3: Multiple lines of computational evidence support a deleterious effect on the gene or gene product; PP4: Patient’s phenotype or family history is highly specific for a disease with a single genetic etiology; PP5: Reputable source recently reports variant as pathogenic, but the evidence is not available to the laboratory to perform an independent evaluation

### Assessment of the pathogenic nature of germline missense variants

Differentiation between rare missense benign variants and pathogenic variants could benefit from the analysis of familial segregation of both variant and phenotype. A clear familial PBMAH presentation has been published for 25 families [21–25, 35–43]. Fifteen variations have been identified in familial PBMAH presentations. Five missense variants segregate with PBMAH phenotype in families: p.(Arg362Trp), p.(Leu365Pro), p.(Leu626Pro), p.(His808Pro) and p.(Leu460Pro).

Recent studies of the prevalence of *ARMC5* germline variants in patients with other adrenal diseases could help to discriminate benign from pathogenic variants [28, 29, 44]. These data, compared to large scale exome and whole genome DNA sequencing studies performed in the genome of the healthy population from different ethnicities, clearly suggest that some frequent variants play no role in PBMAH development. Indeed, some germline missense variants have been more frequently described in the general population than expected for PBMAH causing variations, arguing for their benignity: p.(Ala306Thr), p.(Thr643Met), with a MAF above 0.0001 in addition to p.(Leu156Phe), p.(Gly323Ala), p.(Gln408Arg), p.(Pro559Leu), p.(Pro731Arg) and p.(Gly798Ala) with a MAF above 0.001.

Given the tumor suppressor nature of *ARMC5*, the detection of a second pathogenic event altering *ARMC5* at the somatic tumoral level (such as truncating variants or loss-of-heterozygosity), leading to a bi-allelic impairment of the gene, strongly supports the hypothesis of the deleterious effect of the missense variant identified in leukocyte DNA. We attributed the ACMG criterion PS3 (“well-established in vitro or in vivo functional studies supportive of a damaging effect on the gene or gene product”) to the germline *ARMC5* variants for which one or several second somatic hits have been identified in the adrenal nodules. However, currently, tumoral DNA is not always available because of the frequent use of non-surgical treatment options in PBMAH, such as long-term medical treatment with steroidogenesis inhibitors. Furthermore, copy-number variations (CNV) may be underestimated when using Sanger sequencing method for tumoral DNA.

The occurrence of a second somatic *ARMC5* alteration is strong support for the pathogenic nature of some germline missense *ARMC5* variants: p.(Arg315Trp), p.(Gly323Asp), p.(Asn361Tyr), p.(Arg362Trp), p.(Leu365Pro), p.(Leu460Pro), p.(Leu548Pro), p.(Arg593Trp), p.(Cys657Arg), p.(Pro662His), p.(Ile664Ser), p.(Leu754Pro), p.(Arg764Pro), p.(His808Pro), p.(Arg811Pro), p.(Arg879Trp) and p.(Arg898Trp). On the other hand, no second somatic *ARMC5* alteration has been found after extensive search in patients harboring germline likely benign variants: p.(Leu318Val), p.(Arg502His), p.(Thr643Met) and p.(Arg841His). Similarly, some variants have been identified in non-PBMAH patients: p.(Ala110Asp), p.(Ala500Val), p.(Arg502His), p.(Pro559Leu), p.(Arg611Trp), and p.(Arg682Gln).

Functional studies assessing the loss of apoptotic effect of ARMC5 protein variants, as well as their failed interaction with cullin 3 or substrate proteins, has allowed to assume their pathogenic nature: p.(Arg315Trp), p.(Leu548Pro), p.(Arg593Trp), p.(Cys657Arg), p.(Ile664Ser), p.(Leu754Pro) and p.(Arg898Trp).

At present, no somatic or functional studies have been reported to confirm the pathogenicity of the following 26 missense variants of uncertain significance: p.(Arg60His), p.(Leu136Pro), p.(Val216Met), p.(Arg298His), p.(Arg362Gln), p.(Asp336Gly), p.(Arg357His), p.(Leu394Pro), p.(Thr574Ile), p.(Leu580Pro), p.(Ala590Glu), p.(Arg593Pro), p.(Leu596Arg), p.(Pro608Ser), p.(Arg619Gln), p.(Leu626Pro), p.(Ser669Thr), p.(Ala692Ser), p.(Ala702Glu), p.(Cys734Arg), p.(Val803Gly), p.(Pro826His), p.(Ala838Pro), p.(Ser868Ala), p.(Arg886Pro) and p.(Leu905His); nor of these four likely pathogenic missense variants: p.(Arg362Pro), p.(Pro474Ala), p.(Pro662Leu) and p.(Cys894Trp).

### Somatic *ARMC5* alterations

In PBMAH, DNA sequencing of adrenal nodules reveals different somatic *ARMC5* alterations for a single patient with a single germline *ARMC5* event, in accordance with the Knudson hypothesis for a tumor suppressor gene (Table 2).

**Table 2:** List of the 104 different somatic tumoral *ARMC5* variants, found in PBMAH tissues (or meningioma, when mentioned) and the germline events with which they have been found associated

HGVS cDNA NM_001105247.1	HGVS Protein	Impact	Germline associated variant	Observations	References
cnLOH		Deletion	p.(Gln86*), p.(Arg267*), p.(Asn361Tyr), p.(Arg364*), p.(Leu365Pro), p.(Trp386*), p.(Gln408Arg), p.(Trp476*), p.(Leu548Pro), p.(Ile664Ser), p.(Arg764*), p.(Arg879Trp), p.(Arg898Trp), none		[12–14, 24, 26, 28, 34, 37, 44–46]
cnLOH		Deletion	p.(Arg267*)	Found in meningioma	[26]
Exons 4–6 deletion		Deletion	none		[34]
c.91A>T	p.(Lys31*)	Nonsense	p.(Ala296Cysfs*34)		[12, 13]
c.118del	p.(Leu40*)	Nonsense	large deletion		[12, 13]
c.170dupG	p.(Ile58Asnfs*45)	Frameshift	p.(Ala110Argfs*9)		[22]
c.172del	p.(Ile58Serfs*79)	Frameshift	p.(Arg764*)		[46]
c.205_322del	p.(Pro69Alafs*29)	Frameshift	p.(Gln228*)		[87]
c.210_297del	p.(Ala72Leufs*36)	Frameshift	p.(Ala702_Ser706del)		[12, 13]
c.226C>T	p.(Arg76*)	Nonsense	p.(Ala104Glyfs*7)		[12, 13]
c.231_265del	p.(Ala78Argfs*13)	Frameshift	p.(Cys813Valfs*104)		[34]
c.239C>G	p.(Ala80Gly)	Missense	p.(Ala702_Ser706del)		[12, 13]
c.243_289del	p.(Ser82Valfs*5)	Frameshift	p.(Ala702_Ser706del)		[12, 13]
c.247_256del	p.(Ala83Argfs*51)	Frameshift	p.(His808Pro)		[24]
c.247G>C	p.(Ala83Pro)	Missense	p.(Trp476*)		[45]
c.249_270del	p.(Ser85Profs*45)	Frameshift	p.(Arg315Trp)		[89]
c.261_264del	p.(Gly88Profs*48)	Frameshift	p.(Arg267*)		[46]
c.267del	p.(Gly90Alafs*47)	Frameshift	p.(Gln228*)		[87]
c.276_288del	p.(Pro93Argfs*40)	Frameshift	p.(Arg619*)		[12, 13]
c.279del	p.(Ser94Argfs*43)	Frameshift	p.(Arg654*)		[89]
c.283_286del	p.(Ser95Profs*41)	Frameshift	p.(Arg811Pro)		[82]
c.283_289del	p.(Ser95Argfs*40)	Frameshift	p.(Gln228*)		[87]
c.284C>A	p.(Ser95*)	Nonsense	p.(Arg619*)		[92]
c.290_294del	p.(Ala97Glyfs*4)	Frameshift	p.(Ser779*)		[24]
c.294del	p.(Gly99Glufs*38)	Frameshift	p.(Arg619*)		[92]
c.295G>T	p.(Gly99*)	Nonsense	p.(Asn361Tyr)		[46]
c.306_318del	p.(Pro103Argfs*30)	Frameshift	p.(Leu548Pro)		[46]
c.306_342del	p.(Pro103Argfs*22)	Frameshift	p.(Ala110Argfs*9)		[22]
c.310del	p.(Ala104Profs*33)	Frameshift	p.(Gln228*)		[87]
c.311del	p.(Ala106Argfs*31)	Frameshift	p.(Ala110Argfs*9)		[22]
c.316dup	p.(Ala106Glyfs*13)	Frameshift	p.(Ala110Argfs*9)		[22]
c.319_320del	p.(Ser107Glyfs*11)	Frameshift	p.(Arg898Trp)		[12, 13]
c.325_326delinsT	p.(Pro109Serfs*28)	Frameshift	p.(Gly65Alafs72*)		[37]
c.327del	p.(Ala110Profs*27)	Frameshift	p.(Glu430*), p.(Trp476*)		[12, 13, 45]
c.346del	p.(Ser116Argfs*21)	Frameshift	p.(Trp476*)		[45]
c.347_357del	p.(Ser116Tyrfs*67)	Frameshift	p.(Gln228*)		[87]
c.415T>C	p.(Cys139Arg)	Missense	p.(Arg267*)		[12, 13]
c.420T>A	p.(Cys140*)	Nonsense	p.(Arg267*)		[46]
c.430del	p.(Ala144Argfs*16)	Frameshift	p.(Arg764*)		[46]
c.435C>A	p.(Cys145*)	Nonsense	p.(Arg619*)		[92]
c.456_475 + 5del		Splice	p.(Arg267*)		[12, 13]
c.476-1G>A		Splice	p.(Trp476*)		[45]
c.608del	p.(Ser203Thrfs*2)	Frameshift	p.(Trp476*)		[45]
c.617_845del	p.(Ala206Aspfs*22)	Frameshift	p.(Arg267*)		[12, 13]
c.658del	p.(Leu220Serfs*35)	Frameshift	p.(Leu754Pro)		[13]
c.671C>A	p.(Ala224Glu)	Missense	p.(Arg315Trp)		[89]
c.682C>T	p.(Gln228*)	Nonsense	none		[34]
c.696del	p.(Leu233Trpfs*22)	Frameshift	p.(Arg267*)		[46]
c.703C>T	p.(Gln235*)	Nonsense	p.(Cys657Arg), p.(Arg173*)		[13, 86]
c.789_808del	p.(Glu264Profs*5)	Frameshift	p.(Trp476*)		[45]
c.807C>A	p.(Cys269*)	Nonsense	p.(Trp476*)		[45]
c.913del	p.(Leu305Serfs*9)	Frameshift	p.(Asn361Tyr)		[46]
c.943C>T	p.(Arg315Trp)	Missense	p.(Leu548Pro)		[12, 13]
c.992T>C	p.(Leu331Pro)	Missense	p.(Gly57Glufs*80)		[12, 13]
c.1033C>T	p.(Gln345*)	Nonsense	p.(Trp476*)		[45]
c.1039_1049del	p.(Pro347Glyfs*8)	Frameshift	p.(Arg267*)		[46]
c.1042del	p.(Leu348Trpfs*27)	Frameshift	p.(Arg764*)		[37]
c.1059_1080del	p.(Cys353*)	Nonsense	p.(Trp476*)		[45]
c.1059C>A	p.(Cys353*)	Nonsense	p.(Trp476*)		[45]
c.1084C>T	p.(Arg362Trp)	Missense	p.(Ala110Argfs*9)		[22]
c.1085G>T	p.(Arg362Leu)	Missense	p.(Phe700del)		[12, 13]
c.1158G>A	p.(Trp386*)	Nonsense	p.(Arg267*)		[46]
c.1174del	p.(Ala392Leufs*69)	Frameshift	p.(Arg764*)		[46]
c.1222C>T	p.(Gln408*)	Nonsense	p.(Arg267*)		[46]
c.1297G>T	p.(Glu433*)	Nonsense	p.(Ala110Argfs*9)		[22]
c.1330del	p.(Thr444Profs*17)	Frameshift	p.(Arg593Trp)		[21]
c.1369A>T	p.(Arg457Trp)	Missense	p.(Gln228*)		[88]
c.1474del	p.(Ala492Profs*52)	Frameshift	p.(Cys657Arg)		[13]
c.1507_1508del	p.(Thr503Profs*34)	Frameshift	p.(Ala110Argfs*9)	Found in meningioma	[22]
c.1549G>A	p.(Glu517Lys)	Missense	p.(Arg654*)		[89]
c.1572_1607del	p.(Ala525_Pro536del)	In frame	p.(Arg619*)		[92]
c.1671_1678dup	p.(Gly560Alafs*73)	Frameshift	p.(Arg267*)		[46]
c.1712C>G	p.(Ser571*)	Nonsense	p.(Arg173*)		[86]
c.1746del	p.(Phe583Serfs*47)	Frameshift	p.(Gln86*)		[12, 13]
c.1751T>A	p.(Val584Glu)	Missense	p.(Trp476*)		[45]
c.1777C>T	p.(Arg593Trp)	Missense	p.(Arg267*)		[46]
c.1843C>G	p.(His615Asp)	Missense	p.(Phe14Tyr)		[28]
c.1851del	p.(His518Thrfs*12)	Frameshift	p.(Arg764*)		[38]
c.1855C>T	p.(Arg619*)	Nonsense	p.(Arg267*)		[12, 13, 46]
c.1864G>A	p.(Gly622Arg)	Missense	p.(Arg764*)		[46]
c.1913G>A	p.(Gly638Glu)	Missense	p.(Arg267*)		[46]
c.1960C>T	p.(Arg654*)	Nonsense	p.(Ala110Argfs*9)		[22]
c.1971C>G	p.(Cys657Trp)	Missense	p.(Leu365Pro)		[24]
c.1982T>A	p.(Leu661Gln)	Missense	p.(Arg267*)		[46]
c.2011del	p.(Trp671Glyfs*18)	Frameshift	p.(Arg267*)		[46]
c.2025del	p.(Leu676Trpfs*13)	Frameshift	p.(Gly323Asp)		[82]
c.2029G>T	p.(Glu677*)	Nonsense	p.(Asn361Tyr)		[46]
c.2053_2055del	p.(Leu685del)	In frame	c.476-1G>C		[14]
c.2113del	p.(Leu705Phefs*12)	Frameshift	p.(Gly57Glufs*80)		[13]
c.2116dup	p.(Ser706Phefs*32)	Frameshift	p.(Asn361Tyr)		[46]
c.2123del	p.(Leu708Profs*9)	Frameshift	p.(Ala296Cysfs*34)		[13]
c.2207A>C	p.(Tyr736Ser)	Missense	p.(Arg898Trp)		[12, 13]
c.2228C>T	p.(Ala743Val)	Missense	p.(Trp476*)		[45]
c.2302G>C	p.(Ala768Pro)	Missense	p.(Pro507Leu)		[28]
c.2405C>G	p.(Pro802Arg)	Missense	p.(Trp476*)		[45]
c.2444del	p.(Ala815Leufs*102)	Frameshift	p.(Trp476*)		[45]
c.2486G>A	p.(Gly829Asp)	Missense	none		[34]
c.2522G>A	p.(Arg841His)	Missense	p.(Arg315Trp)		[89]
c.2525T>A	p.(Phe842Tyr)	Missense	p.(Arg654*)		[89]
c.2542G>T	p.(Glu848*)	Nonsense	p.(Arg619*)		[92]
c.2599G>T	p.(Glu867*)	Nonsense	p.(Arg619*)		[38]
c.2611G>T	p.(Glu871*)	Nonsense	p.(Asn361Tyr)		[46]
c.2647C>T	p.(His883Tyr)	Missense	p.(Phe14Tyr)		[28]
c.2666T>A	p.(Leu889Gln)	Missense	p.(Gln408Arg)		[28]
c.2734G>A	p.(Glu912Lys)	Missense	p.(Arg267*)		[46]
c.2755del	p.(Ala919Leufs*6)	Frameshift	p.(Arg267*)		[46]

cnLOH, copy-neutral loss of heterozygosity

Initially, *ARMC5* locus was first identified by the evidence of 16p LOH in PBMAH [12]. Somatic 16p copy neutral LOH has been reported in several studies in adrenocortical nodules from PBMAH patients [12–14, 24, 26, 34, 37, 44–46]. Apart from 16p LOH, numerous other somatic *ARMC5* genetic alterations have been observed. In total, 104 different somatic events (Table 2) have been described: LOH, 51 SNV (27 missense variants, 23 nonsense variants, and 2 SNV affecting splice sites), 51 indels (49 frameshift deletions or insertions, 2 in frame deletions) and 1 large deletion of exons 4–6) (Table 2 and Fig. 2B). Interestingly, 12 of these somatic variants (p.(Ile58Asnfs*45), p.(Pro109Serfs*28), p.(Gln228*), p.(Arg315Trp), p.(Gln345*), p.(Leu348Trpfs*27), p.(Arg362Trp), p.(Trp386*), p.(Arg593Trp), p.(Arg619*), p.(Arg654*) and p.(Arg841His)) have also been described as first germline events (Tables 1 and 2).

Taking into account the tumor suppressor gene model of *ARMC5*, extra-adrenal tumoral DNA has been analyzed for bi-allelic *ARMC5* alterations. Besides PBMAH, *ARMC5* alterations affecting both alleles have been implicated in the development of meningiomas: p.(Thr503Profs*34) somatic event added to the germline already known p.(Ala110Argfs*9) variant in a first patient [22]; tumoral loss of heterozygosity (LOH) along with the germline p.(Arg267*) variant in another patient [26]. In the current literature, besides these two cases with demonstrated somatic *ARMC5* alterations, meningiomas have been reported in 10 other *ARMC5* patients [21, 23–25, 27] and in two Korean sisters reported before the identification of *ARMC5* [47]. Meningioma is, at present, the only other tumor type besides PBMAH, for which molecular studies suggest a causative role for *ARMC5*. Somatic tumoral *ARMC5* alterations were not identified in breast, parathyroid, thyroid tumors or pancreatic NET from PBMAH patients with a germline *ARMC5* pathogenic variant [22, 38].

## Genotype/phenotype correlation

A more pronounced phenotype has been reported in patients with PBMAH and germline *ARMC5* pathogenic variant than in wild-type patients [13, 20, 48], with regard to the intensity of Cushing’s syndrome according to the usual endocrine and clinical markers of cortisol excess: elevated 24-h urinary free cortisol, morning plasma cortisol after 1 mg DST, and midnight plasma cortisol, associated with a more suppressed ACTH. Consequently, PBMAH patients present more often with diabetes and high blood pressure requiring the use of antihypertensive medications. Their adrenal glands appear larger than in wild-type patients, and harbor more numerous nodules. Therefore, *ARMC5*-mutated patients are more frequently treated than wild-type patients, whether by adrenalectomy or by steroidogenesis inhibitors in order to control Cushing’s syndrome. A recent study from our group reported the largest series of patients with PBMAH to date, including a broad range of phenotypes, from bilateral adrenal incidentaloma without any clinical evidence of endogenous cortisol excess to massively enlarged adrenals associated with severe hypercortisolism and showed that, additionally to the bilateral adrenal nodules, *ARMC5*-mutated patients constantly present with a cortisol dysregulation, at least with a mild autonomous cortisol secretion [20]. In keeping with these observations, the study stated that *ARMC5* genotyping should be offered to all index patients presenting clear bilateral adrenal macronodules associated with at least a mild autonomous cortisol secretion defined by a morning plasma cortisol after 1 mg DST above 50 nmol/L as previously proposed [48, 49] and to all first-degree relatives of *ARMC5* pathogenic variant carriers [20]. For index-cases, the negative predictive value of these simple criteria is 100%, meaning that no *ARMC5*-mutated patient should be missed, while the positive predictive value is close to 20% [20] which is higher than various genetic screening for other types of endocrine tumors (pituitary, parathyroid). Furthermore, additional genetic screening should be considered in specific situations. Indeed, in 2021, two teams have identified *KDM1A* germline pathogenic variants as the molecular cause of hereditary PBMAH associated with food-dependent Cushing’s syndrome [50, 51], a very rare presentation of PBMAH due to the illegitimate expression of the glucose-dependent insulinotropic polypeptide receptor (GIPR) in adrenocortical cells, reinforcing the idea that PBMAH is a genetic disease [52]. *KDM1A* genotyping should then be proposed to all patients with a suspicion of food-dependent cortisol secretion (*i.e.,* low fasting plasma cortisol, increasing after meals or oral glucose test) [1, 53–55]. In the case of associated clinical features suggesting a syndromic presentation such as MEN1, FAP or HLRCC, a complementary genotyping of *MEN1*, *APC* or *FH* genes should be considered [1]. However, with the increasing use of multigenic panel in routine practice one should consider to include all these gene in a PBMAH panel.

In most of the papers describing the clinical presentation of *ARMC5* mutated and wild-type PBMAH patients, ethnic origin is not mentioned. But PBMAH due to *ARMC5* germline alteration is probably spread all over the world, since there are many reports from North and South America, Europe, Middle East and Asia; and in our own experience in patients from African ancestry.

If *ARMC5*-mutated patients harbor a distinct phenotype compared to wild-type PBMAH patients, we can also speculate that truncating variants could be more deleterious than missense variants and associated with a more severe form of the disease. Accordingly, 56% of patients with truncating *ARMC5* variants are primarily referred to clinical departments in front of overt signs of Cushing’s syndrome, versus only 21% of patients with non-truncating pathogenic variants, for which the incidental diagnosis in abdominal imaging is far more frequent [20]. This could be explained, at least in part, by the significantly higher values of midnight plasma cortisol in patients with truncating variants (nonsense and frameshift with premature stop codon) compared to non-truncating missense variants, witnessing for a perceptible increased cortisol output in these patients [20]. However, the other biological parameters are not statistically different, maybe due to the limited number of patients with *ARMC5* variant. Likewise, this approach was used to compare variants affecting the different domains of the ARMC5 protein, but no significant difference could be detected [20]. These observations raise pending questions about the pathogenic mechanisms of ARMC5 proteins altered by amino acids substitutions, which should be addressed in further basic and clinical investigations. ARMC5 protein has not been successfully crystallized at this point, but this could be a major help for the interpretation of missense variants.

The penetrance of PBMAH in *ARMC5* pathogenic variant carriers seems high but incomplete in the reported familial cases [23, 24] but remains largely unknown at present, mostly because of the rarity and the late onset of the disease. Similarly, the affected relatives are often younger than the index case and appear with a more attenuated disease compared to the index case. However, the mechanisms causing this phenotypic variability have not been studied to date and would need extensive familial investigations.

PBMAH is often associated with aberrant response of cortisol secretion to various stimuli, through the illegitimate expression of G-protein coupled receptors in adrenocortical cells, such as GIP receptor associated with food-dependent Cushing’s syndrome [56, 57], LH/hCG receptor associated with post-menopausal Cushing’s syndrome [58], beta-adrenergic receptors associated with a cortisol response to upright posture, stress or sport [59], vasopressin receptors associated with cortisol response to upright posture and vasopressin agonists [60], serotonin receptors [61], and other less common receptors. Illegitimate expression of beta-adrenergic or vasopressin receptors has already been described in *ARMC5* variant carriers [12, 13, 23]. In contrast, illegitimate expression of the GIP receptor has never been reported in *ARMC5* patients [13]. This was suggestive of a potential different molecular predisposition, which was later elucidated in 2021 with the identification of *KDM1A* as the genetic cause of PBMAH associated with food-dependent Cushing’s syndrome [50, 51].

The presence of a germline *ARMC5* variant is strongly correlated with typical histopathological patterns of PBMAH subtype 1 according to Violon and colleagues [62], associating large coalescent yellow nodules composed of 70–90% of clear cells, 10–30% of compact cells and < 10% of oncocytic cells, with the presence of round fibrous septa within the macronodules. Residual internodular adrenal is unfrequently observed. Immunohistochemistry usually shows strong positivity for HSD3B2 in clear cells and CYP17A1 in compact cells.

## *ARMC5* function: in vitro data and animal models

*ARMC5* inactivation results in apparently contradictory effects: an impaired apoptosis and a decreased steroidogenesis. Indeed, on one hand, the pro-apoptotic effect of wild-type *ARMC5* transfected in adrenocortical carcinoma cell line H295R is lost when the cells are transfected with the *ARMC5* mutants [12, 13], likely to explain the adrenal tumorigenesis. On the other hand, *ARMC5* silencing by siRNA or shRNA in H295R leads to a decreased steroidogenesis associated with a reduced expression of steroidogenic enzymes, as observed in PBMAH primary cell cultures from *ARMC5* mutated patients [12, 14]. However, the effects of *ARMC5* mutants’ overexpression on steroidogenesis have not been assessed. Altogether, despite the altered steroidogenesis, the uncontrolled tumorigenesis resulting from the impaired apoptosis is likely to explain a global excessive cortisol output by the adrenals [63].

ARMC5 protein comprises a C-terminal BTB (Broad-Complex, Tramtrack and Bric-à-Brac) domain, which allows the interaction with the E3-ubiquitine ligase cullin 3, leading to ARMC5 ubiquitination and further degradation by the proteasome. Our group recently showed that an *ARMC5* missense variant affecting its BTB domain results in a loss of the interaction with cullin 3 and then in a stabilization of ARMC5 protein [15]. Moreover, it has just been demonstrated that ARMC5 also regulates the degradation of other proteins [64–68]. Indeed, ARMC5 acts as an adaptor for Cullin 3 complex by recruiting protein substrates including RPB1 [65, 67], SREBF [66, 68] and NRF1 [64]. Interestingly, *ARMC5* p.R593W variant located in the intermediate region fails to interact with RPB1 protein, leading to the accumulation of RPB1 protein in mutated PBMAH tissues [65].

Two *Armc5* deficient murine models have been generated: the complete knock-out *Armc5*-/- is lethal in most cases between days 6.5 and 8.5 of embryonic development [17] but the rare living pups are smaller than the wild-type ones and the aged mice develop a non-nodular adrenal hyperplasia along with high corticosterone levels [18] and have a higher risk of neural tube defects [67]. The heterozygous knock-out mice *Armc5* ± show a normal development and present with transient low corticosterone levels at 1 year which could result from an impaired steroidogenesis, followed by a normalization and even an increased corticosteronemia at 18 months in one third of cases, this could be consecutive to a raise of ACTH levels in response to the initial low corticosteronemia [17], without adrenal nodule nor hyperplasia, in accordance with the two-hit model of *ARMC5*-driven adrenocortical tumorigenesis, which cannot result from haploinsufficiency.

## Discussion

The pathogenic nature of truncating *ARMC5* variants is rarely a matter of debate but the assessment of germline missense variants is far more delicate and rests on a beam of arguments comprising their frequency in general population, in silico predictions, the identification of somatic *ARMC5* variants in the tumoral DNA from operated patients, familial segregation, and in vitro studies for some patients. In our experience, considering that functional in vitro studies of *ARMC5* function are difficult to standardize for routine diagnosis, the occurrence of a second somatic hit in the adrenal nodules is strong evidence for the pathogenic nature of a germline missense variant. It is not clear whether or not this feature covers the definition of the PS3 ACMG criterion (“well-established in vitro or in vivo functional studies supportive of a damaging effect on the gene or gene product”), but we assume that this should be considered for tumor-suppressor genes with a proven two-hit inactivation model that are usually not found as pure somatic alterations in sporadic tumors.

These criteria and their interpretation are framed by the five classes of the ACMG guidelines [33], which have been primarily designed for classical Mendelian diseases and genes. But the field is constantly evolving, thanks notably to genome-wide association studies, revealing new insights in the high complexity of the relation between genetics and diseases [69], which may not be entirely reflected by the five ACMG classes. This led many researchers to suggest amendments to the classification, which will be still subject to continuous refinements. For instance, the distinction between disease-causing and disease-predisposing genes has been recently proposed, based on the observations in chronic pancreatitis [70].

The available data are not sufficient to classify some missense variants, notably because many PBMAH patients do not undergo adrenal surgery and thus, tumor samples are now rarely available for somatic genotyping. Functional studies, so far, are limited to the demonstration of the loss of the apoptotic effect of the wild-type ARMC5 by some missense variants [12, 13] and loss of ARMC5 interaction with cullin-3 or its protein substrates by missense variants in the BTB domain or in the intermediate domain [15, 65]. The other missense alterations might also lead to a loss of *ARMC5* function but functional studies need to demonstrate this hypothesis. A better understanding of *ARMC5* physiological role is required to develop systematic functional studies of *ARMC5* variants to corroborate the loss of function induced by the genetic alterations observed in PBMAH patients.

Until recently, all the somatic pathogenic variants identified were observed in patients having also a germline *ARMC5* variant. This is different from other tumor suppressor genes, which can be found mutated only in tumor DNA as a pure somatic alteration in sporadic diseases. This suggests that *ARMC5* alterations, with at least haploinsufficiency, need to be present early in adrenal development to promote the onset of PBMAH. However, a recent study reported 5 complete gene deletions, 1 partial deletion (exons 4–6) and 2 SNV affecting *ARMC5* at the somatic level in 6 PBMAH patients without germline *ARMC5* variation [34]. These very interesting findings would need to be corroborated in further studies. Conversely, no concomitant *CTNNB1, PRKACA, PRKAR1A* or *GNAS* somatic variant (classically met in cortisol-producing adenomas [71–79]) with a germline *ARMC5* alteration has been yet reported to our knowledge. The occurrence of different second somatic alterations in multiple adrenal nodules of the same patient is also a quite unique feature of the tumor suppressor gene *ARMC5*. It should be noted also that bi-allelic germline inactivation of *ARMC5* – whether by homozygosity or compound heterozygosity – has not been reported so far, suggesting that it might be lethal during embryonic development, as observed in murine models.

*ARMC5* germline and somatic alterations have been associated with the occurrence of meningiomas in several patients, including familial cases. But the real incidence of meningiomas in *ARMC5* mutated patients remains unknown and needs to be extensively investigated, since no systematic brain imaging in these patients have been reported to date. Apart from meningiomas, there is no clear association between *ARMC5* genetic alterations and sporadic nor familial other tumor type. This suggests that despite a rather ubiquitous expression of *ARMC5* [80], its inactivation can promote tumorigenesis in only few tissues.

*ARMC5* variants account for around 20% of PBMAH index cases, and *KDM1A* for less than 5%. Therefore, 75% or more of PBMAH index cases have no identified molecular cause at present, which is a challenging field of future research.

## Conclusion

This work is the first extensive analysis of all the *ARMC5* genetic alterations reported so far in the literature with an addition of 36 unpublished variants identified in our center. A total of 238 different *ARMC5* alterations are reported here: 146 identified on germline DNA and 104 on tumoral DNA (12 reported both as germline and somatic alterations). *ARMC5* pathogenic variants are spread through the entire coding sequence of the gene. There is no clear hotspot region although some amino acids can be more frequently altered.

The present study is an important source of information and provides a list of variants, classified upon their pathogenic nature, which could help clinicians and geneticists to discriminate pathogenic from benign variants.

This list of variants would benefit from additional data from the various centers worldwide performing *ARMC5* genotyping routinely, and could result in the creation of a public and evolving database, as it has already been developed for *TP53* (https://tp53.isb-cgc.org/), compiling the whole *ARMC5* germline and somatic variants, as well as the available experimental data and anonymized clinical information of variant carriers, both index and related cases, allowing to increase and share the knowledge about the genotype/phenotype correlation of this challenging disease.

## Data Availability

All data generated or analyzed during this study are included in this published article.
